# t-BHQ Provides Protection against Lead Neurotoxicity via Nrf2/HO-1 Pathway

**DOI:** 10.1155/2016/2075915

**Published:** 2015-12-21

**Authors:** Fang Ye, Xiaoyi Li, Lili Li, Jing Yuan, Jun Chen

**Affiliations:** Department of Occupational and Environmental Health and Ministry of Education Key Lab for Environment and Health, School of Public Health, Tongji Medical College, Huazhong University of Science and Technology, Wuhan 430030, China

## Abstract

The neurotoxicity of lead has been well established, and oxidative stress is strongly associated with lead-induced neurotoxicity. Nrf2 is important for protection against oxidative stress in many disease models. We applied t-BHQ, which is an Nrf2 activator, to investigate the possible role of Nrf2 in the protection against lead neurotoxicity. t-BHQ significantly attenuated the oxidative stress in developmental rats by decreasing MDA level, as well as by increasing SOD activity and GSH content, in the hippocampus and frontal cortex. Furthermore, neuronal apoptosis was detected by Nissl staining, and Bax expression was inhibited in the t-BHQ-treated group. Results showed that t-BHQ suppressed ROS production and caspase 3/7 activity but increased intracellular GSH content, in SH-SY5Y cells under lead exposure. Moreover, *in vivo* and *in vitro*, t-BHQ enhanced the nuclear translocation of Nrf2 and binding to ARE areas but did not induce Nrf2 transcription. These phenomena were confirmed using RT-PCR, EMSA, Western blot, and immunofluorescence analyses. Subsequent upregulation of the expression of HO-1, NQO1, and GCLC was observed. However, knockdown of Nrf2 or HO-1 adversely affected the protective effects of t-BHQ against lead toxicity in SH-SY5Y cells. Thus, t-BHQ can protect against lead neurotoxicity, depending on the Nrf2/HO-1 pathway.

## 1. Introduction

Lead poisoning remains a major public health concern despite extensive initiatives to reduce environmental lead exposure caused by the wide availability of this toxic heavy metal. Lead induces numerous adverse health effects that are dose-dependent and partly irreversible. Lead even targets multiple organ systems, of which the nervous system appears to be the most sensitive and chief target [1]. Although lead damages the neural system regardless of age, young children are particularly vulnerable to its toxic effects. Low-level lead exposure has been suggested to significantly affect the IQ, behavior, concentration ability, and attentiveness of a child [2, 3].

Lead toxicity may be mediated by several mechanisms. The effect of lead on oxidant/antioxidant imbalance has been reported as a major mechanism of lead-induced toxicity. Lead catalyzes oxidative reactions and generates reactive oxygen species (ROS). Methane dicarboxylic aldehyde (MDA), which is a clinical marker of oxidative stress, such as lipid peroxidation, increases [4] and the reduced glutathione (GSH) content decreases in the brain when the neural system is exposed to lead [5]. In addition, several important antioxidant enzymes, such as GSH reductase (GR), superoxide dismutase (SOD), and catalase (CAT) [6], are inactivated by lead. Lead-induced ROS accumulation inhibits the production of sulfhydryl antioxidants, causes inflammation, damages nucleic acids, inhibits DNA repair, inhibits enzyme reactions, impairs heme production, and initiates lipid peroxidation in cellular membranes [7, 8].

Antioxidants, such as vitamin E, olive leaf, N-acetyl-cysteine (NAC), and plant extracts, have been extensively examined to remedy lead toxicity [9–12]. NAC was found to remarkably decrease MDA levels, normalize reduced GSH/oxidized GSH ratios, improve the GSH status in the brain and liver, and increase cell survival rates. The antioxidant effect of NAC is also effective in reducing and reversing the oxidant effect of increased levels of aminolevulinic acid, which is a marker of symptomatic lead toxicity [13]. Vitamins B, C, and E can chelate lead from tissues and restore the oxidant/antioxidant balance [14]. Therefore, administration of various antioxidants can prevent or subdue various toxic effects of lead and production of oxidative stress.

In the present study, we focused on the nuclear factor (erythroid-derived 2)-like 2 (Nrf2) considering that the imbalance of oxidant/antioxidant is a major mechanism of lead toxicity. Nrf2 is a transfactor and regarded as one of the most important mechanisms in the cell for protection against oxidative stress. Nrf2 belongs to a subset of basic leucine zipper genes, sharing a conserved structural domain that belongs to the Cap“N”Collar subfamily of basic leucine zipper transcription factors [15]. Nrf2 under normal or unstressed conditions is tethered in the cytoplasm by the Keap1 protein [16]. Keap1 can target Nrf2 for ubiquitination by CUL3 ligase and subsequent degradation by the proteasome [17]. Activated Nrf2 translocates into the nucleus, where it binds to a small Maf protein and activates the transcription of target genes known as phase II enzymes, such as heme oxygenase-1 (HO-1), NAD(P)H: quinine oxidoreductase 1 (NQO1), and glutamate cysteine ligase catalytic subunit (GCLC). HO-1 is a rate-limiting enzyme for degrading heme into biliverdin, carbon monoxide (CO), and free iron [18]. HO-1 and its reaction products protect against various injuries [19]. Nrf2 or HO-1 knockdown in mice results in substantially increased susceptibility of mice to numerous chemical toxic and disease conditions associated with oxidative pathology [20]. By contrast, enhancing Nrf2 or HO-1 by genetic tools or some antioxidants has beneficial effects on cell survival. Administration of curcumin or luteolin protects against As^3+^ toxicity or traumatic brain injury (TBI) through an Nrf2-dependent mechanism [21, 22].

The protective effect of Nrf2 against numerous hazard materials or diseases has been extensively investigated. However, only a few studies have focused on lead neurotoxicity. In addition, some antioxidants, such as curcumin and vitamin E, which have been proven to exhibit lead toxicity, can activate the Nrf2/HO-1 pathway, but whether these agents depend on the Nrf2/HO-1 pathway to protect against lead toxicity remains unclear [23, 24]. Thus, in the present study, we investigated the role of Nrf2 in protection against lead toxicity* in vivo* and* in vitro*. We applied tert-butylhydroquinone (t-BHQ), which has been considered as an Nrf2 and phase II detoxification enzyme inducer, to explore the capability of Nrf2/HO-1 to protect against lead neurotoxicity [25, 26]. t-BHQ is a metabolite of the antioxidant butylated hydroxyanisole, which can cross the blood-brain barrier, and it has been demonstrated to show neuroprotective effects in several disease models [27]. t-BHQ has been found to be beneficial for the nervous system because of its protective effects against TBI, amyloid *β*, and 1-methyl-4-phenylpyridinium (MPP^+^) toxicity through the Nrf2/HO-1 pathway [28–30]. However, the protective effects of t-BHQ against lead toxicity remain unknown. Thus, we also investigated the beneficial effects of t-BHQ against lead toxicity.

## 2. Method

### 2.1. Chemicals and Materials

t-BHQ, lead acetate (PbAc), cycloheximide (CHX), and actinomycin D (Act.D) were purchased from Sigma-Aldrich (CA, USA). DMEM/F12, PBS (pH 7.4), and fetal bovine serum were from GIBCO (Invitrogen, Carlsbad, CA, USA). Deionized water was produced by an ultrapure water purification system.

### 2.2. Animals and Experimental Treatments

Male Sprague-Dawley rats (approximately 4-5 weeks old; weight, 60–70 g) were obtained from the Animal Experiment Center Of Wuhan University and housed in the Animal Experiment Center of Huazhong University of Science and Technology. All animal experiments were performed according to the guidelines for the care and use of laboratory animals by National Institutes of Health. Before dosing, rats were housed in stainless steel cages in an air-conditioned room at a constant temperature of 25°C ± 2°C and acclimatized for 4 days. The room was lit from 6:00 a.m. to 6:00 p.m. and alternated with 12 h of darkness. Subsequently, 40 animals were randomly divided into eight groups according to treatment: con (corn oil), t-BHQ, corn oil + PbAc (3 subgroups 10, 30, and 60 mg/kg), and t-BHQ + PbAc (3 subgroups: 10, 30, and 60 mg/kg). Animals received t-BHQ dissolved in corn oil at 150 mg/kg or corn oil only once daily for 8 days by oral gavage. On day 4, test groups of rats were injected intraperitoneally with 10, 30, or 60 mg/kg lead acetate once daily for 5 days. The rats were then sacrificed. The brains were removed, washed in ice-cold saline, and dissected into different brain regions, namely, the cortex and hippocampus. Doses used in this study were based on previous studies on the neuroprotection of t-BHQ in brain injury models and lead poisoning models [27, 31]. Solutions of t-BHQ and lead acetate were freshly prepared every day.

### 2.3. MDA and GSH Contents, as well as SOD Activity, in the Hippocampus and Frontal Cortex

*In vivo*, 20 mg of hippocampus tissues or 50 mg of frontal cortex tissues was homogenized on ice in PBS and centrifuged for 10 min at 12000 rpm at 4°C. The supernatant was collected.* In vitro*, SH-SY5Y cells were washed and collected from plates. The cells were resuspended in PBS, directly immersed in liquid nitrogen, and rapidly thawed by agitating the suspensions twice in a water bath at 37°C. The cells were centrifuged for 10 min at 12000 rpm and 4°C. The supernatant was collected and analyzed.

MDA level, GSH content (Nanjing Jiancheng Biochemistry Co., Nanjing, China), and SOD activity (Dojindo Laboratories, Japan) were measured using a microplate reader (Bio-Tec, CA, USA) according to the manufacturer's instructions. Protein concentrations were determined by BCA protein assay kit (Pierce, Rockford, IL, USA) as described by the manufacturer.

### 2.4. Nissl Stain

Animals were sacrificed, and brain tissues were immediately perfused with saline followed by ice-cold 4% paraformaldehyde phosphate buffer overnight. The tissues were then embedded in paraffin and cut into 4 *μ*m coronal sections with a vibratome (Leica, Germany). Coronal sections were washed by distilled water, stained with 0.5% thionine for 10 min at 50°C, and differentiated in 95% ethyl alcohol for 15 min. The tissues were dehydrated in 100% alcohol for 5 min twice, cleared in xylene for 5 min twice, and mounted by resinous medium for quantification. Neurons were counted in the frontal cortex and hippocampus CA1 region. The percentage of damaged neurons was calculated by dividing the number of damaged neurons by the total number of neurons [32].

### 2.5. Cell Viability

Cell viability of SH-SY5Y cells (ATCC, USA) was assessed by the Cell Counter Kit-8 (CCK-8) assay (Dojindo Laboratories, Japan) according to the manufacturer's instruction. SH-SY5Y cells purchased from the Chinese Type Culture Collection (Shanghai, China) were seeded in a 96-well plate and left to attach overnight. After the indicated treatments, 10 *μ*M CCK-8 solution dissolved by serum-free medium was added to each well. The cells were incubated for 1 h in an incubator, and absorbance was quantified on an automated microplate reader (Bio-Tec, CA, USA).

### 2.6. Caspase 3/7 Activity in Cells

Caspase 3/7 activity was measured with commercial kits (AAT Bioquest, CA, USA). Cells planted in the 96-well plate received indicated treatments and then reacted with DEVD-AMC substrate in the reaction buffer. The cells were incubated at room temperature for 1 h in the dark. The cell plate was centrifuged at 800 rpm for 2 min and then monitored by an automated microplate reader (Bio-Tec, CA, USA) at Ex/Em = 350 nm/450 nm. The results were normalized to the numbers of cells present to obtain the average caspase 3/7 activity.

### 2.7. ROS Detection in Cells

Flow cytometry was used to analyze intracellular ROS by the fluorescence probe 2′7′-dichlorodihydrofluorescein diacetate (DCFH-DA) (Beyotime Institute of Biotechnology, Ltd., Shanghai, China). SH-SY5Y cells were seeded into six-well plates. At the end of treatment, the medium was replaced with fresh serum-free medium containing 10 *μ*g/mL DCFH-DA. Cells were incubated for 30 min at 37°C in the dark and then washed three times with serum-free medium. Collected cells were then washed twice with cold PBS and analyzed immediately by flow cytometry (Becton Dickinson, San Jose, CA, USA). A total of 10,000 cells were analyzed for each sample, and the mean fluorescence intensity was obtained.

### 2.8. SiRNA Transfection

Predesigned siRNA for human Nrf2 (Santa Cruz, CA, USA), human HO-1, and negative control siRNA (Invitrogen, CA, USA) were transfected into SH-SY5Y cells by Lipofectamine RNAi max (Invitrogen, CA, USA) according to the manufacturer's protocol. The siRNAs (60 pmol) were incubated in six-well plates with transfection reagent for 10 min at room temperature to allow the formation of transfection complexes, which were then used for transformation. After 8 h of transfection, cells were changed to fresh medium and then subjected to various treatments as described.

### 2.9. RT-PCR

RNA was extracted from the hippocampus, frontal cortex, or SH-SY5Y cells with TRIzol reagent (Invitrogen, Carlsbad, CA, USA) according to the manufacturer's protocol. The quality and quantity of RNA were measured by NanoDrop 1000 (Thermo Scientific, USA). Approximately 2 *μ*g of extracted total RNA was used for cDNA synthesis using Omniscript RT kit (Thermo Scientific, USA) in accordance with the manufacturer's instructions. RT-PCR was performed in 10 *μ*L containing 100 nM primers (Table 1) purchased from Invitrogen (CA, USA) and SYBR Green PCR Master Mix (Invitrogen, Carlsbad, CA, USA). Amplification was conducted in an ABI 7900HT sequence detection system. PCR conditions were as follows: 95°C for 2 min, 40 cycles at 95°C for 15 s, and 60°C for 60 s. Each mRNA expression was normalized to human GAPDH or rat GAPDH mRNA expression using the comparative cycle threshold method. Identity and purity of the amplified product were checked by analyzing the melting curve plotted at the end of amplification.

### 2.10. Western Blot

Total proteins were extracted using ice-cold RIPA buffer (Beyotime Institute of Biotechnology, Ltd., Shanghai, China) containing protein inhibitor cocktail (Roche, Germany). Cytoplasmic and nuclear proteins were prepared. Cells or tissues were first lysed or homogenized using cytoplasmic protein lysis buffer [10 mM HEPES-NaOH (pH 7.9), 10 mM KCl, 1 mM EDTA, 1 mM DTT, and 0.2% NP-40] on ice. The lysates were then ultracentrifuged at 10,000 rpm for 10 min at 4°C. The supernatants were collected as cytoplasmic protein. The pelleted nuclei were resuspended in nuclear protein lysis buffer (20 mM HEPES-NaOH, 420 mM NaCl, 1 mM EDTA, 1 mM DTT, and 10% NP-40). Lysates were centrifuged after 40 min of incubation at 4°C. The supernatants containing nuclear proteins were obtained. Protein concentrations were determined by BCA protein assay kit (Pierce, Rockford, IL, USA) as described by the manufacturer.

Equivalent amounts of protein were separated by 10% Sodium Dodecyl Sulfate- (SDS-) polyacrylamide gel electrophoresis and transferred to NC membranes (Millipore Co., Billerica, MA, USA). The membranes were blocked in 5% non-fat milk in TBST [10 mM Tris-HCl (pH 7.6), 0.1% Tween 20] for 1 h at room temperature, followed by incubation with either one of the following primary antibodies at 4°C overnight: Nrf2, NQO1, Bax, and Bcl2 (Santa Cruz, CA, USA); HO-1 (Abcam, CA, USA); and *β*-actin and PCNA (Sungene Biotech, Tianjin, China). The immunoblots were then incubated with species-appropriate secondary antibody conjugated with horseradish peroxidase (Abbkine, CA, USA) for 1 h at room temperature. The membranes were developed using an electrochemiluminescence (ECL) kit (Pierce, Rockford, IL, USA) according to the manufacturer's protocol. Signals were detected by a chemiluminescence detection system (Systemgen, England). The density of the immunoreactive bands was analyzed using ImageJ 1.41 (National Institutes of Health, USA).

### 2.11. Immunofluorescence

SH-SY5Y cells were plated on glass cover slips into 24-well plates for 1 day and then treated with t-BHQ or DMSO for various times. After treatment, cells were fixed for 30 min in 4% paraformaldehyde, permeabilized with 10% triton, and blocked with goat serum for 30 min. Cells were then incubated overnight with Nrf2 antibody (1 : 50, Santa Cruz, CA, USA) at 4°C. Cover slips were washed with PBS (5 min, three times) and incubated with anti-rabbit Alexa Fluor 488 (1 : 200, Molecular Probes, Netherlands) for 1 h at room temperature. Finally, the cover slips were stained with DAPI (Roche, Germany) for 5 min. The images were then acquired with a fluorescence microscope (Olympus, Japan).

### 2.12. Electrophoretic Mobility Shift Assay (EMSA)

Synthetic double-stranded oligonucleotide (Beyotime Institute of Biotechnology, Ltd., Shanghai, China) containing the Nrf2 binding domain (ARE) was labeled with biotin. The ARE sequence was 5′-ACT GAG GGT GAC TCA GCA AAA TC-3′. Chemiluminescent Nucleic Acid Detection Module Kit (Pierce, Rockford, IL, USA) was used to determine ARE-binding activity. Nuclear extract (7.5 *μ*g) was incubated on ice for 15 min in gel shift binding buffer. DNA-protein complexes were resolved by 6.5% polyacrylamide gel electrophoresis at 100 V for 1 h and then transferred to nylon membrane. The membrane was blocked, conjugated, washed, and substrated by corresponding solution after cross-linking for 10 min by a UV-light cross-linking instrument. Finally, resultant signals were visualized using an enhanced ECL kit (Pierce, Rockford, IL, USA), and the density of the bands was analyzed using ImageJ 1.41 (National Institutes of Health, USA).

### 2.13. Statistical Analysis

All experiments were repeated at least three times. Data were expressed as means ± SEM. The results were analyzed using one-way ANOVA with* post hoc* Dunnett's or LSD's test, and a *P* value of <0.05 was considered statistically significant.

## 3. Results

### 3.1. t-BHQ Alleviates PbAc-Induced Alterations in Redox Status in the Hippocampus and Frontal Cortex

**Figure 1:**
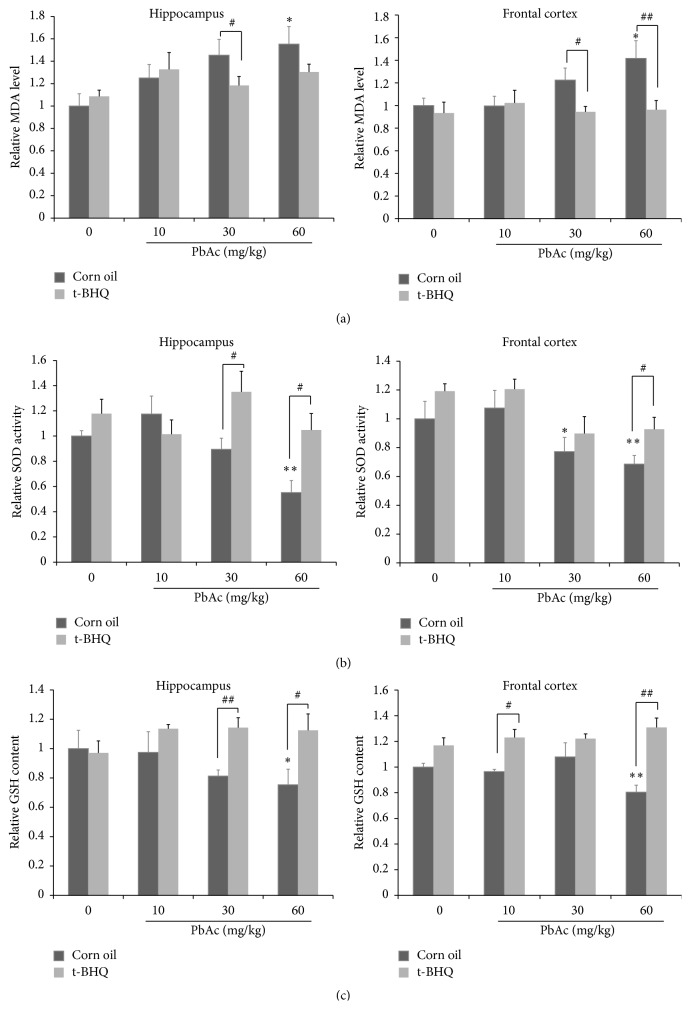
t-BHQ alleviates PbAc-induced alterations in redox status in the hippocampus and frontal cortex. Rats pretreated with t-BHQ (150 mg/kg) or corn oil for three days by oral gavage then cotreated with varies doses (10, 30 or 60 mg/kg) PbAc for five days by intraperitoneal injection (ip). The levels of MDA (a), SOD activity (b), and GSH content (c) were detected in these eight groups in the hippocampus and frontal cortex. *n* = 5 in each group. Data represent the mean ± SEM of the results. ^*∗*^ *P* < 0.05 and ^*∗∗*^ *P* < 0.01 represent significant differences compared with the control group (corn oil without PbAc). ^#^ *P* < 0.05 and ^##^ *P* < 0.01 represent significant differences between two groups with or without t-BHQ treatment at the same concentration of PbAc.

The effects of t-BHQ on rats exposed to PbAc were evaluated in detail. The hippocampus and frontal cortex, which have been declared as major areas influenced by lead, were separated and detected independently [33]. The redox status contains two aspects, namely, oxidant stress and antioxidant reserves. We investigated the MDA level, as an indicator of oxidant stress, as well as SOD activity and GSH content as antioxidant reserves. Elevated MDA was detected in the corn oil group in a lead dose-dependent manner; the level increased by 55% or 41% at 60 mg/kg PbAc in the hippocampus or frontal cortex, respectively. Administration of t-BHQ significantly reduced MDA generation and maintained the MDA level similar to that of the control in the brain when rats were exposed to various doses (10, 30, or 60 mg/kg) of PbAc (Figure 1(a)). By contrast, SOD activity and GSH content decreased in a dose-dependent manner after PbAc exposure (Figures 1(b) and 1(c)). At 60 mg/kg PbAC, SOD activity and GSH content decreased by approximately 45% and 28% in the hippocampus and by 32% and 20% in the frontal cortex, respectively. By contrast, t-BHQ upregulated SOD activity and inhibited the loss of GSH in the brain. Therefore, our data confirmed that t-BHQ was neuroprotective against PbAc. Moreover, 60 mg/kg PbAc treatment exhibited the maximum neurotoxicity, so this dose was selected for the following experiments.

### 3.2. t-BHQ Promotes Nrf2 Nuclear Translocation and Induces Phase 2 Enzyme Expression in the Hippocampus and Frontal Cortex

**Figure 2:**
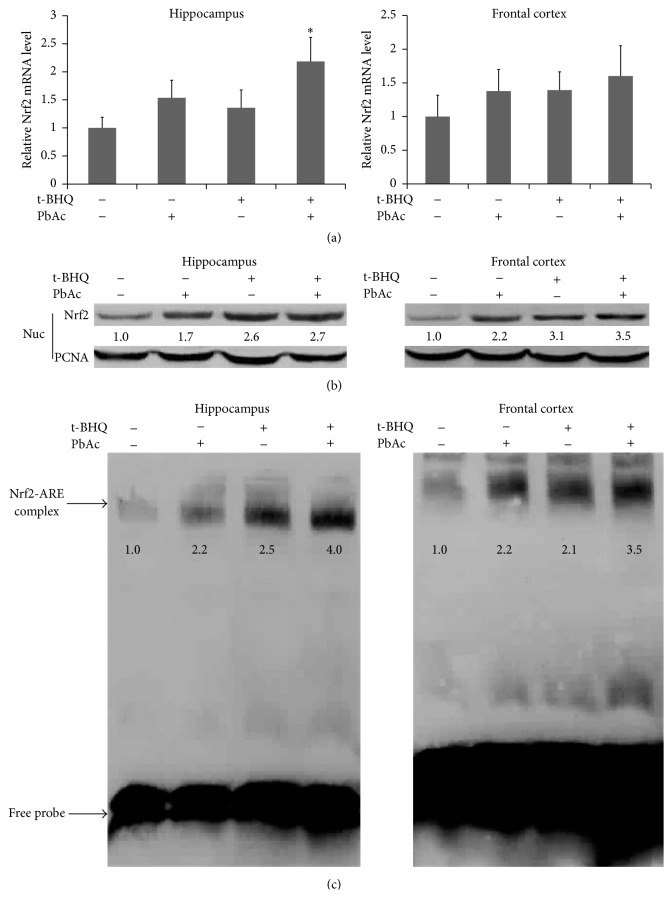
t-BHQ promotes Nrf2 nuclear translocation but does not induce Nrf2 transcription in the hippocampus and frontal cortex. (a) Nrf2 mRNA level was quantified by RT-PCR and the appropriate dose of PbAc (60 mg/kg) was chosen in this experiment. *n* = 5 in each group. (b and c) Nuclear protein was isolated and applied in Western blot experiment (b) and EMSA experiment (c). Nrf2-ARE binding activity and the content of Nrf2 in nucleus were detected and the immunoblots were quantified. *n* = 5 in each group. Data represent the mean ± SEM of the results. ^*∗*^ *P* < 0.05 represents significant differences compared with the control group.

We also determined the effect of t-BHQ on the Nrf2-antioxidant system in the brain of developmental rats. We examined the mRNA level of Nrf2 in four groups (control, PbAc, t-BHQ, and PbAc + t-BHQ). t-BHQ and PbAc did not affect Nrf2 transcription in the hippocampus and frontal cortex. However, Nrf2 mRNA was induced in the PbAc + t-BHQ group in the hippocampus (Figure 2(a)). EMSA results showed that t-BHQ enhanced nuclear protein Nrf2 binding to AREs in the brain, which was consistent with the Western blot results. This phenomenon indicated that Nrf2 protein accumulated in the nucleus (Figures 2(b) and 2(c)). PbAc treatment also enhanced Nrf2 concentration in the nucleus as a self-defense reaction to PbAc exposure. These findings indicated that t-BHQ could promote Nrf2 translocation from the cytoplasm to nucleus, resulting in elevated binding ability to AREs and induced downstream gene expression.

**Figure 3:**
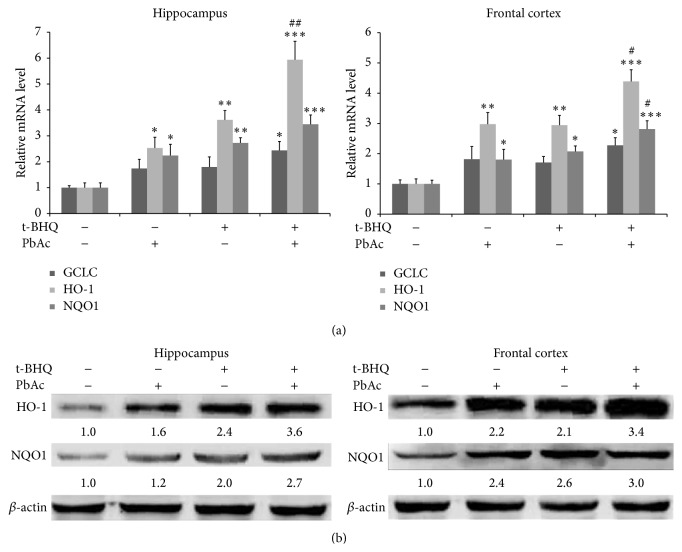
t-BHQ upregulates the expression of Nrf2 downstream targets in the hippocampus and frontal cortex. Rats were pretreated with t-BHQ or corn oil and then subjected to PbAc exposure (60 mg/kg). The levels of mRNAs and protein were examined by RT-PCR and Western blot, respectively. The mRNA of GCLC, HO-1, and NQO1 (a) and the protein levels of HO-1 and NQO1 (b) were analyzed in both the hippocampus and frontal cortex. *n* = 5 in each group. Data represent the mean ± SEM of the results. ^*∗*^ *P* < 0.05, ^*∗∗*^ *P* < 0.01, and ^*∗∗∗*^ *P* < 0.001 represent significant differences compared with the control group. ^#^ *P* < 0.05 and ^##^ *P* < 0.01 represent significant differences between two groups with or without t-BHQ treatment at the same concentration of PbAc (60 mg/kg).

Three important genes (HO-1, NQO1, and GCLC) regulated by Nrf2 were examined. RT-PCR results showed that HO-1 and NQO1 mRNA levels were upregulated after PbAc treatment, unlike GCLC (Figure 3(a)). Additionally, administration of t-BHQ further enhanced HO-1 and NQO1 expression compared with that in the PbAc-treated group but did not induce GCLC (Figure 3(a)). t-BHQ upregulated the protein expression of HO-1 and NQO1, which was consistent with the changes in mRNA (Figure 3(b)). These results suggested that t-BHQ could upregulate the mRNA and protein levels of Nrf2 downstream genes in the brain.

### 3.3. Pb-Induced Neuronal Apoptosis Could Be Attenuated by t-BHQ in the Hippocampus and Frontal Cortex

**Figure 4:**
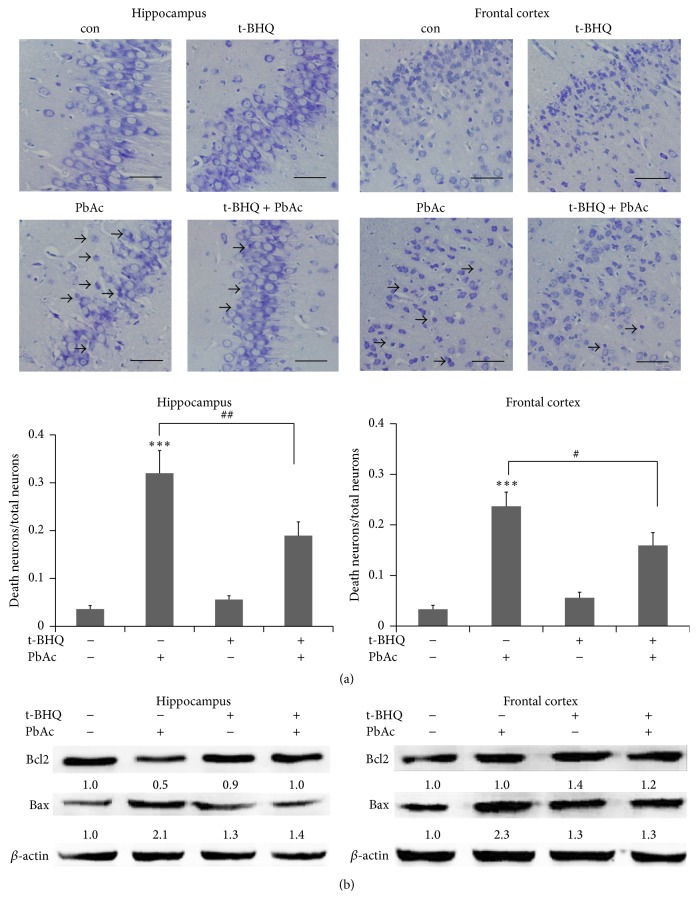
Administration of t-BHQ protects rats against PbAc toxicity. (a) Nissl staining of rat brain sections was applied to investigate the histomorphology of cells in the hippocampus CA1 region and frontal cortex. The injured neurons (arrows) were characterized by cytoplasmic shrinkage, nuclear pyknosis, or hyperchromasia. Scar bar = 50 *μ*m. The ratios of death neuroes/total neuroes were caculated and analyzed in these two regions, respectively. Data were collected from four fields of view. (b) The proapoptotic protein of Bax and antiapoptotic protein of Bcl2 were detected in the hippocampus and frontal cortex; immunoblots were quantified, respectively. *n* = 5 in each group. Data represent the mean ± SEM of the results. ^*∗∗*^ *P* < 0.01 and ^*∗∗∗*^ *P* < 0.001 represent significant differences compared with the control group. ^#^ *P* < 0.05 and ^##^ *P* < 0.01 represent significant differences between two groups with or without t-BHQ treatment at the same concentration of PbAc (60 mg/kg).

Nissl staining was applied to evaluate neuron viability in the hippocampus CA1 region and frontal cortex. Rats unexposed to PbAc in the control and t-BHQ groups did not exhibit any damage or change in neuronal morphology. By contrast, dead neurons were observed in PbAc-exposed rats. However, t-BHQ treatment resulted in lower neuron loss and decreased apoptosis ratio from 32% to 19% in the hippocampus and from 24% to 16% in the frontal cortex (Figure 4(a)). Quantitative results showed that t-BHQ administration could significantly attenuate PbAC-induced neuron death in the hippocampus CA1 region and frontal cortex. Consistent with Nissl staining results, t-BHQ restrained the increase in Bax and decrease in Bcl2 caused by PbAc in the brain (Figure 4(b)). Hence, t-BHQ could significantly inhibit Pb-induced neuronal apoptosis in the brain because Bax and Bcl2 are two important proteins associated with apoptosis [34].

### 3.4. t-BHQ Activates Nrf2 and Induces HO-1 Expression in an Nrf2-Dependent Manner in SH-SY5Y Cells

**Figure 5:**
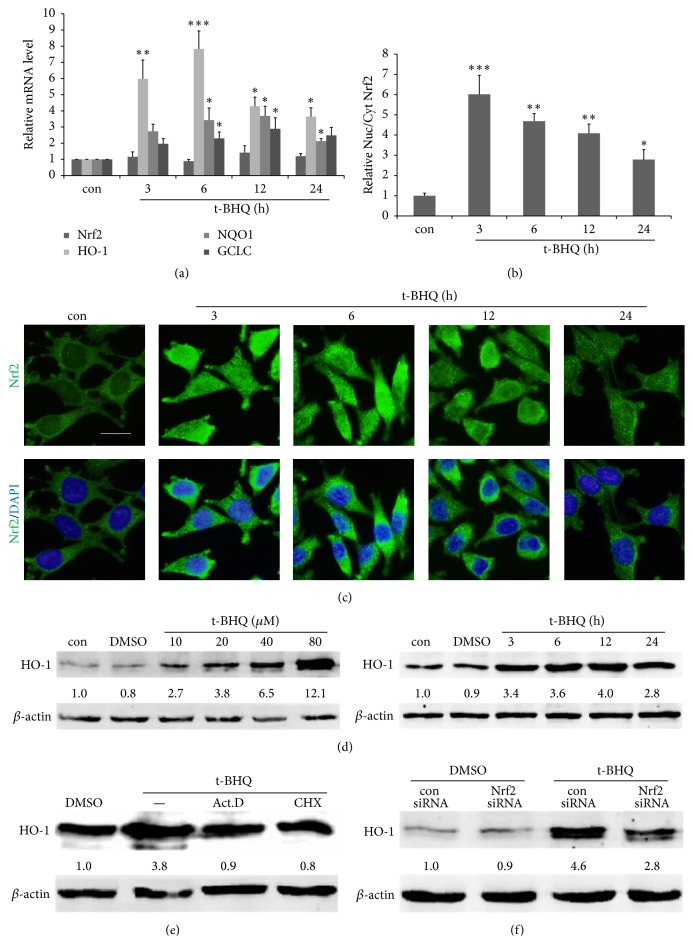
t-BHQ promotes Nrf2 tranlocation from cytoplasm to nucleus and induces the Nrf2-regulated gene expression, especially HO-1. (a) the mRNAs of HO-1, NQO1, GCLC, and Nrf2 were detected by RT-PCR, when cells were treated by t-BHQ (40 *μ*M) for various time (3, 6, 12, or 24 h). (b and c) The immunofluorescent assay was applied to investigate Nrf2 transloction from cytoplasm to nucleus. Scar bar = 20 *μ*m. The content of Nrf2 in nucleus and cytoplasm was analyzed by ImageJ and the ratio of nue/cyt was caculated. *n* = 3 in each group. (d) Cells were treated by t-BHQ at various doses (10, 20, 40, and 80 *μ*M) or for various time (3, 6, 12, and 24 h); the protein of HO-1 was detected by Western blot. (e) Cells were pretreated with Act.D (0.5 *μ*g/mL) or CHX (10 *μ*g/mL) for 1 h and then treated with t-BHQ or DMSO for 12 h. Cellular proteins were extracted for HO-1 detection. (f) Cells were transfected with 60 pmols control siRNA or Nrf2 siRNA for 24 h and then treated with t-BHQ (40 *μ*M) for an additional 12 h; the HO-1 protein was detected by immunoblot. Data represent the mean ± SEM of the results. ^*∗*^ *P* < 0.05, ^*∗∗*^ *P* < 0.01, and ^*∗∗∗*^ *P* < 0.001 represent significant differences compared with the control group.

We treated SH-SY5Y cells with 40 *μ*M t-BHQ based on previously published results and present findings on the performance of high-dose t-BHQ on toxicity and induced cell apoptosis (data not shown) [35, 36]. t-BHQ did not induce Nrf2 transcription as detected by RT-PCR (Figure 5(a)), which was consistent with the results* in vivo*. Moreover, Nrf2 immunofluorescence showed the nuclear translocation of Nrf2 from the cytoplasm. The nucleus/cytoplasm ratio of Nrf2 peaked at 3 h after t-BHQ treatment (Figures 5(b) and 5(c)). Activation of Nrf2 induced the gene transcription of HO-1, NQO1, and GCLC. HO-1 mRNA was upregulated by sevenfold at 6 h compared with the control (Figure 5(a)). Immunoblot analysis of SH-SY5Y cells treated with t-BHQ showed a time- and dose-dependent upregulation of HO-1 protein (Figure 5(d)). The highest induction was observed at 12 h. In related experiments, protein synthesis inhibitor CHX and transcription inhibitor Act.D failed to demonstrate t-BHQ-mediated upregulation in HO-1 protein, indicating that HO-1 was synthesized* de novo* (Figure 5(e)). In addition, knockdown of Nrf2 by transfection of Nrf2-siRNA reduced the ability of t-BHQ-induced HO-1 upregulation (Figure 5(f)). Thus, the results implied that induction of HO-1 by t-BHQ was mainly regulated by Nrf2, which could bind to AREs and induce HO-1 expression. These results suggested that the antioxidant t-BHQ activated the Nrf2/HO-1 pathway.

### 3.5. t-BHQ Protects against PbAc-Induced ROS Production and Apoptosis through the Nrf2/HO-1 Pathway

**Figure 6:**
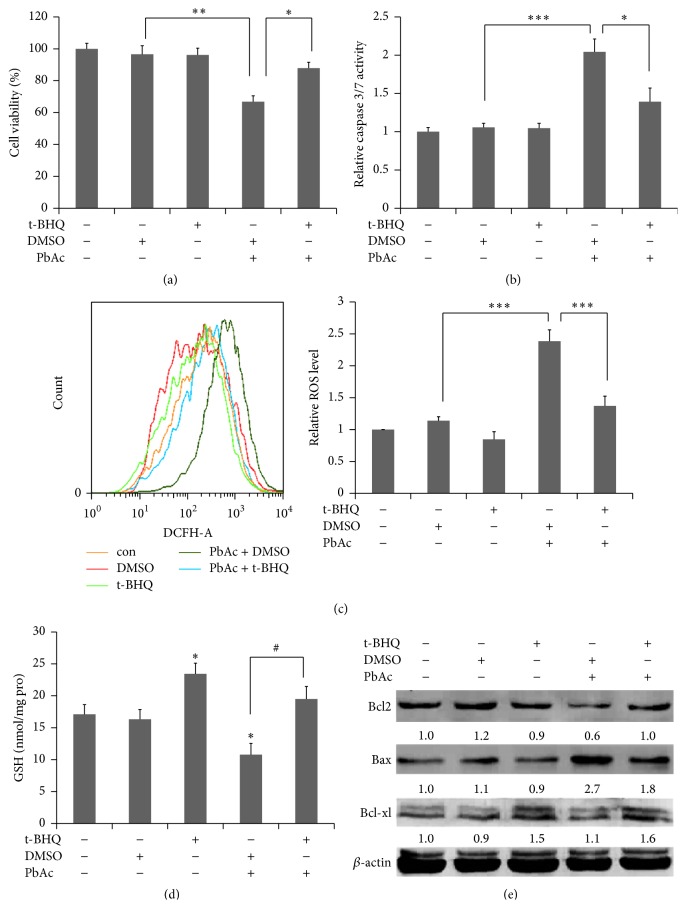
t-BHQ confers protection against PbAc-induced oxidative stress and apoptosis. The SH-SY5Y cells were pretreated with t-BHQ (40 *μ*M) or DMSO for 12 h, followed by PbAc exposure (25 *μ*M) for 24 h. Cell viability (a), caspase 3/7 activity (b), ROS production (c), GSH content (d), and apoptotic related proteins of Bcl2, Bax, and Bcl-xl (e) were analyzed. All data represent the mean ± SEM of the results. ^*∗*^ *P* < 0.05, ^*∗∗*^ *P* < 0.01, and ^*∗∗∗*^ *P* < 0.001 represent significant differences.

We investigated cell viability in SH-SY5Y cells using CCK8 to examine the effect of t-BHQ on apoptosis and cell survival. Treatment of cells with 25 *μ*M PbAc led to decreased viability (66%) compared with the control (Figure 6(a)). The PbAc dose of 25 *μ*M was selected because cell viability at this concentration was approximately 71% (data not shown). In related experiments, PbAc enhanced endogenous caspase 3/7 activity 2.1-fold, indicating increased apoptosis (Figure 6(b)). Pretreatment of t-BHQ suppressed cell death and caspase 3/7 activity, which exhibited a protective role against PbAc toxicity (Figures 6(a) and 6(b)). Immunoblot analysis of proteins showed PbAc-induced upregulation of Bax and downregulation of Bcl2 and Bcl-xl induced by PbAc. These phenomena were prevented by t-BHQ pretreatment (Figure 6(e)). Our results provided evidence that t-BHQ reversed PbAc-induced ROS production, which is one of the important mechanisms of lead toxicity (Figure 6(c)). In addition, t-BHQ increased GSH content and reversed the depletion of GSH caused by PbAc (Figure 6(d)). These results significantly suggested that t-BHQ could protect against the oxidative stress and apoptosis induced by PbAc.

**Figure 7:**
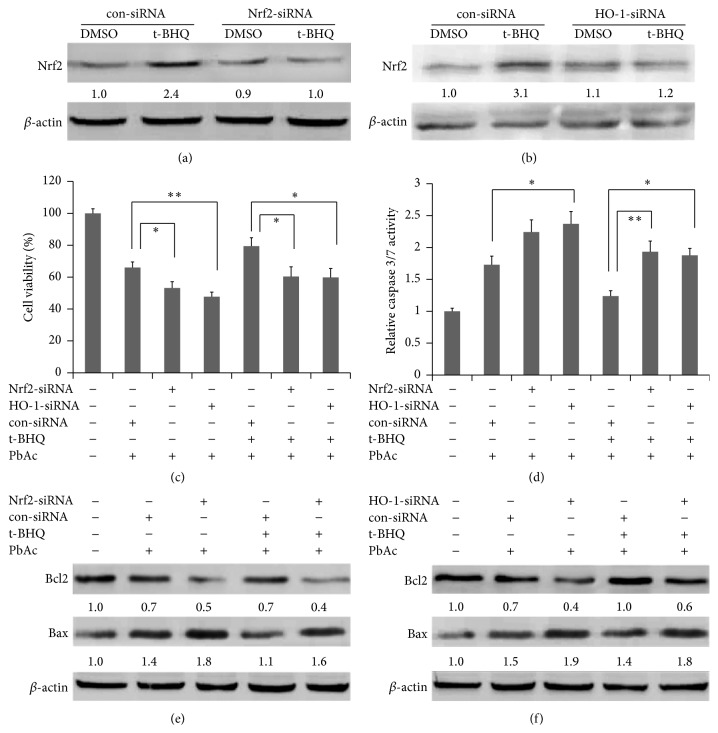
Nrf2/HO-1 pathway mediates the protection of t-BHQ against lead toxicity. (a) and (b) con-siRNA, Nrf2-siRNA, or HO-1-siRNA were transfected into SH-SY5Y cells for 12 h before treatment with t-BHQ. The potein levels of Nrf2 and HO-1 were analyzed by Western blot. (c–f) Cells were transfected with con-siRNA, Nrf2-siRNA, or HO-1-siRNA for 12 h and treated with t-BHQ or DMSO for another 12 h and then exposed to PbAc for 24 h in the presence of t-BHQ or DMSO as indicated. Cell viability (c) and caspase 3/7 activity (d) were measured and analyzed. Western blot analyzed the proapoptotic protein of Bax and antiapoptotic protein of Bcl2 in cells transfected with Nrf2-siRNA (e) or HO-1 siRNA (f) and then treated with t-BHQ (40 *μ*M) and PbAc (25 *μ*M) as indicated above. All data represent the mean ± SEM of the results. ^*∗*^ *P* < 0.05 and ^*∗∗*^ *P* < 0.01 represent significant differences.

Nrf2 and HO-1 were silenced using Nrf2 and HO-1 siRNAs, respectively, to investigate the specific role of the Nrf2/HO-1 pathway in t-BHQ-mediated cell protective effect against PbAc (Figures 7(a) and 7(b)). SH-SY5Y cells were transfected with siRNA of the control, Nrf2, or HO-1, followed by treatment with DMSO or t-BHQ. The cells were then exposed to PbAc and finally analyzed by CCK8 and caspase 3/7 activity. The results revealed that knockdown of Nrf2 or HO-1 resulted in increased sensitivity of cells to PbAc. Lower cell survival and higher caspase 3/7 activity were observed in the Nrf2-siRNA + PbAc and HO-1-siRNA + PbAc groups compared with those in the con-siRNA + PbAc group (Figures 7(c) and 7(d)). Silencing Nrf2 or HO-1 significantly abolished the protective effect of t-BHQ against PbAc, although t-BHQ treatment induced cell survival and inhibited caspase 3/7 activation (Figures 7(c) and 7(d)). Hence, immunoblot was used to detect Bax and Bcl2 proteins when Nrf2 and HO-1 were silenced. Knockdown of Nrf2 or HO-1 resulted in upregulated Bax protein and downregulated Bcl2 protein in cells and attenuated the beneficial effects of t-BHQ against PbAc treatment (Figures 7(e) and 7(f)). These results showed that the Nrf2/HO-1 pathway played a significant role in t-BHQ-induced apoptosis reduction.

## 4. Discussion

We investigated the neuroprotection of t-BHQ through the Nrf2/HO-1 pathway against lead toxicity in the brain of developmental rats and SH-SY5Y cells.* In vivo* and* in vitro* results confirmed that t-BHQ attenuated lead toxicity depending on the Nrf2/HO-1 pathway.

Lead remains a significant global concern because of its neurotoxic effect [37]. Oxidative stress is considered an important mechanism of lead toxicity and regulates many cell signaling pathways and cell survival [7, 38]. MDA level, which is one of the products of lipid peroxidation and commonly used as a biomarker of oxidative damage and membrane injury, is strongly correlated with lead concentration in the brain of exposed rats [39]. In the present study, we observed that the brain MDA level increased in a dose-dependent manner in PbAc-treated rats, and ROS production increased in PbAc-treated SH-SY5Y cells. In addition, PbAc decreased SOD activity and GSH content in the hippocampus and frontal cortex. GSH and antioxidative enzymes protect the brain from oxidative damage; their expression and activity have been demonstrated to be critical in modulating the response of neurons to various kinds of stress and cell survival [40, 41]. Imbalance between the oxidation and reduction states caused by lead can lead to cell stress and even cell death. In our investigation on apoptotic markers, we obtained data showing that PbAc induced caspase 3/7 activation in SH-SY5Y cells and histological changes in the brain of PbAc-treated rats. In a related experiment, we also found that PbAc downregulated Bcl2 expression and upregulated Bax expression* in vivo* and* in vitro*, and these proteins are associated with apoptosis. PbAc can induce apoptosis in several brain areas, including the hippocampus and frontal cortex [42], through the upregulation of proapoptotic factors (e.g., caspases, Bax, and Bcl-2). In cultured cells, 10 *μ*M Pb promoted apoptotic cell death in the PC12 cell line [43].

Lead chelators, such as CaNa_2_EDTA and dimercaptosuccinic acid [7], and antioxidants, such as NAC and olive leaf [11, 13], were identified and examined to antagonize lead toxicity. NAC was found to decrease MDA levels, improve GSH status, normalize reduced GSH/oxidized GSH ratios, and induce cell survival in brain and liver tissues in rats exposed to lead. Consistent with the effects of NAC, t-BHQ abolished apoptosis and maintained the balance between the oxidation and reduction states* in vivo* and* in vitro*. t-BHQ is a member of antioxidants that have potentially beneficial effects against lead neurotoxicity. The protective role of t-BHQ has been verified in many reports [44]. t-BHQ showed neuroprotective effects on Paraquat-induced dopaminergic cell degeneration in C57BL/6 mice and in PC12 cells, as well as against apoptosis in amyloid *β*-injected rats [29, 36]. The protective effects of t-BHQ have been attributed to the activation of Nrf2, which is an important translation factor in the maintenance of cellular homeostasis.

t-BHQ increases the level of Nrf2 mediated by a posttranscriptional mechanism, rather than an increase in Nrf2 mRNA levels* in vivo* and* in vitro*. Consistent with curcumin, t-BHQ does not induce Nrf2 transcription but depresses its degradation such that Nrf2 accumulates in the cell [21]. Increased Nrf2 dissociates from keap1 protein, translocates into nucleus, binds to the ARE area, and induces the expression of many antioxidant and phase 2 metabolizing enzymes [45]. The present results confirmed the mechanisms of t-BHQ protective effects mentioned above. Additionally, the data suggested that t-BHQ led to Nrf2 nuclear activation and accumulation, as well as induced HO-1 and NQO1, but not GCLC, in the hippocampus and frontal cortex. Enhanced expression of HO-1 and NQO1, as well as increased mRNA level of GCLC, was observed in the SH-SY5Y cells. GCLC constitutes the enzyme glutamate cysteine ligase (GCL) with GCLM, and GCL mainly mediates GSH synthesis [46]. We noticed that t-BHQ increased GCLC transcription and GSH content in SH-SY5Y cells, but not in the brain of rats. However, some studies showed that the level of GCLC mRNA increases both in primary cortical astrocytes and in mice [47, 48]. The different results* in vivo* are probably due to the different conditions used in the studies.

We found that t-BHQ increased HO-1 expression mediated by Nrf2, considering that HO-1 is regulated by several translation factors, such as Nrf2 and NF*κ*B, but mainly by Nrf2 [49]. The Nrf2/HO-1 axis constitutes the essential part of the conserved cellular defense against a wide array of endogenous and exogenous stresses. In this study, we tested the role of the Nrf2/HO-1 pathway in the prevention of lead-induced oxidative stress and cell death. Knockdown of Nrf2 or HO-1 resulted in higher sensitivity of cells to lead exposure as shown by increased cell death and higher caspase 3/7 activity. These data suggested that the Nrf2/HO-1 pathway had beneficial effects against lead neurotoxicity. These effects of Nrf2/HO-1 have been observed in many aspects. Nrf2 knockout mice were more sensitive to 1-methyl-4-phenyl-1,2,3,6-tetrahydropyridine, which could induce Parkinson's disease-like lesions in mice [50]. HO-1-deficient mice succumbed to unfettered oxidative tissue injury [51]. Nrf2/HO-1 exhibits the important ability to protect cells and plays an indispensable role in the immune system. Several natural plant extracts, such as curcumin and zeaxanthin, also show beneficial effects against oxidative damage through Nrf2 activation [21, 52]. Given that t-BHQ could protect against lead neurotoxicity* in vivo* and* in vitro*, as well as activating the Nrf2/HO-1 pathway, whether the protective effects of t-BHQ were mediated through the Nrf2/HO-1 pathway remains unknown. Silencing Nrf2 or HO-1 significantly increased caspase 3/7 activity and Bax expression, as well as decreasing cell survival rate and Bcl2 expression, in cells pretreated with t-BHQ and exposed to PbAc. These results indicated that t-BHQ protected against lead toxicity mainly through the Nrf2/HO-1 pathway. In the study of Lee et al., in which Oligonucleotide Microarray Analysis was used to analyze primary cortical astrocytes and Nrf2 knockout mice, most of the t-BHQ-induced genes, such as HO-1, NQO1, and GCLC, were reportedly dependent on Nrf2. Thus, Nrf2/HO-1 activation has been proven to mediate t-BHQ beneficial effects on cell survival* in vivo* and* in vitro* [28–30]. Knockdown of Nrf2 or HO-1 abolished the effects of t-BHQ on inducing these protective genes in SH-SY5Y cells. Therefore, the protective effects of t-BHQ were subdued. However, further studies should be performed to determine whether t-BHQ alleviates lead neurotoxicity* in vivo* through the Nrf2/HO-1 pathway. Application of Nrf2-knocked out mice is necessary, which is a limitation of the current study.

In conclusion, our preliminary study identified a new antioxidant, t-BHQ, which protects against oxidative stress and cell death caused by lead toxicity* in vivo* and* in vitro*. Furthermore, the beneficial effects of t-BHQ were mainly mediated by the Nrf2/HO-1 pathway.

## Figures and Tables

**Table 1:** Primer pairs used in q-PCR.

Gene	Primer sequence	
Human Nrf2	F	5′-ATT GCC TGT AAG TCC TGG TCA-3′
	R	5′-ACT GCT CTT TGG ACA TCA TTT CG-3′

Human GCLC	F	5′-CAA GGA CGT TCT CAA GTG GG-3′
	R	5′-CAT ACT CTG GTC TCC AAA GG-3′

Human HO-1	F	5′-AAC TTT CAG AAG GGC CAG GT-3′
	R	5′-CTG GGC TCT CCT TGT TGC-3′

Human NQO1	F	5′-CGC AGA CCT TGT GAT ATT CCA G-3′
	R	5′-CGT TTC TTC CAT CCT TCC AGG-3′

Human GAPDH	F	5′-CTG ACT TCA ACA GCG ACA CC-3′
	R	5′-TGC TGT AGC CAA ATT CGT TGT-3′

Rat Nrf2	F	5′-GGT TGC CCA CAT TCC CAA AC-3′
	R	5′-GGC TGG GAA TAT CCA GGG C-3′

Rat GCLC	F	5′-CCA CTG TCC AAG GTT GAC GA-3′
	R	5′-TTG CTA CAC CCA TCC ACC AC-3′

Rat HO-1	F	5′-GCG AAA CAA GCA GAA CCC A-3′
	R	5′-GCT CAG GAT GAG TAC CTC CCA-3′

Rat NQO1	F	5′-ATT GTA TTG GCC CAC GCA GA-3′
	R	5′-GAT TCG ACC ACC TCC CAT CC-3′

Rat GAPDH	F	5′-CAA GTT CAA CGG CAC AGT CAA-3′
	R	5′-TGG TGA AGA CGC CAG TAG ACT C-3′
